# Methanol fixation of plant tissue for Scanning Electron Microscopy improves preservation of tissue morphology and dimensions

**DOI:** 10.1186/1746-4811-9-36

**Published:** 2013-10-02

**Authors:** Mark J Talbot, Rosemary G White

**Affiliations:** 1 Commonwealth Scientific and Industrial Research Organisation, Division of Plant Industry, Canberra, ACT 2601, Australia

**Keywords:** Critical point drying, Plant cell morphology, *Arabidopsis thaliana*, *Hordeum vulgare*, *Gossypium hirsutum*

## Abstract

**Background:**

It is well known that preparation of biological (plant and animal) tissues for Scanning Electron Microscopy (SEM) by chemical fixation and critical point drying results in shrinkage of tissues, often by up to 20-30%, depending on the tissue type and fixation protocol used. We sought to identify a protocol that would preserve tissue size and morphology better than standard chemical fixatives and dehydration regimes. We compared a range of processing techniques by quantifying changes in tissue size and recording details of surface morphology using leaf tissues from three commonly studied species; *Arabidopsis thaliana*, barley and cotton.

**Results:**

All processing protocols altered tissue dimensions. Methanol fixation and dehydration, followed by a further short (1 h) dehydration step in ethanol and critical point drying (which was based on a previously published method), preserved tissue dimensions most consistently of all protocols tested, although it did cause 8% shrinkage in all three species. This protocol was also best for preservation of surface morphology in all three species. We outline a recommended protocol and advise that the method is best trialled for different tissues, especially thicker or larger samples.

**Conclusions:**

This study shows that simultaneous fixation and dehydration in methanol followed by ethanol results in better preservation of dimensions and morphology of critical point dried plant tissues than other fixation and dehydration procedures. It is a quick and simple method, and requires standard SEM preparation equipment.

## Background

A well-known artefact of preparing biological tissue for Scanning Electron Microscopy (SEM) is the shrinkage of tissue during fixation, dehydration and critical point drying (CPD) steps. In the past, these artefacts were documented largely by comparing the surface morphology of animal and some plant tissues prepared using different protocols. Several studies showed that after ‘conventional’ fixation with glutaraldehyde, dehydration with ethanol followed by CPD, plant and animal tissues can shrink to approximately 67-75% of their original size, measured as either volume, length, or width [1-3]. The total change in size arises from cumulative shrinkage in each processing step, particularly the dehydration step [2,4], and varies with different specimens [1].

Earlier investigations into the causes of tissue shrinkage found that chemical fixation was not critical for good preservation [2,4]. In fact, Boyde and Boyde (1980) showed that unfixed potato tuber tissue processed through dehydration and CPD shrank less (15%) than glutaraldehyde fixed tissue (30%; [2]). Another source of shrinkage was the critical point drying process and post-CPD storage, which could be due to retention of water or ethanol in the tissue after drying, which then slowly evaporated during storage [2,5].

Given that omission of aqueous fixation might improve preservation of plant tissue [2] we sought to identify a routine procedure that would maintain tissue size and morphology for SEM examination. Although cryo-fixation (plunging into liquid nitrogen or propane) has been shown to be optimal for preservation of morphology [6], it requires dedicated, expensive equipment and results in an approximately 9% increase in tissue volume due to ice crystal formation during freezing [6,7]. Also, once prepared, the tissue cannot be stored for future imaging. Freeze-drying is superior to CPD for preserving animal tissue dimensions (e.g., [5]), but for plant [8,9] and fungal [7] tissues it gives results similar to glutaraldehyde-fixation and CPD-processing at best, presumably because these tissues contain considerably more water.

Fixation and dehydration of plant tissue with methanol has been reported to improve preservation compared to conventional techniques [10,11]. This method has been used to document tissue morphology (e.g., [12-14]) and for epidermal cell size analysis [15] by SEM, and also to preserve roots for widefield light microscope imaging [16]. Methanol processing was shown to have little or no effect on coleoptile dimensions, whereas conventional formaldehyde-acetic acid-alcohol (FAA) or glutaraldehyde fixation caused tissue shrinkage [10]. However, the effect of methanol processing on dimensions of critical point dried tissue for SEM has not been investigated. This is important when using SEM for quantitative analysis, for example, of cell size [15].

This study aimed to identify an SEM preparation method that preserves close to original tissue morphology and dimensions. A protocol based on solvent fixation is an attractive alternative to conventional SEM fixatives since tissue is simultaneously fixed and dehydrated [10] and can then be transferred directly to the CPD. We trialed methanol and other organic solvents (ethanol and acetone) as alternatives to conventional SEM fixatives and quantified changes in tissue dimensions after processing through CPD. We provide a detailed comparison of conventional fixation methods with solvent-based protocols and conclude that the methanol-ethanol protocol is generally the best for preservation of plant tissues for SEM.

## Results

To identify a robust SEM preparation method that caused the least modifications to tissue dimensions and morphology we compared the effects of seven different fixation protocols on the preservation of leaf samples from *Arabidopsis thaliana*, barley and cotton. The first two fixatives, FAA and 3% glutaraldehyde, are routinely used for SEM. Published methods using these fixatives were followed with little or no modification to provide a basis for comparison with the five solvent-based fixation procedures we tested. Since 70% ethanol is a quick, relatively non-toxic fixative used for a variety of purposes, including fixation of samples in the field, we included this protocol. We were particularly interested in testing the methanol-based protocols recommended from previous work [10]. Methanol fixation was followed by either further dehydration in methanol and critical point drying in methanol (‘Methanol’), or by ethanol dehydration and critical point drying in ethanol (‘Methanol-ethanol’). We also investigated 100% ethanol and acetone as fixatives since these solvents are commonly used to dehydrate tissues for SEM and Transmission Electron Microscopy (TEM). Treatment effects were monitored by expressing the final area of processed leaves or leaf pieces as a percentage of the area of unfixed material, and by imaging leaf morphology.

### Effect of fixation methods on tissue area

**Figure 1:**
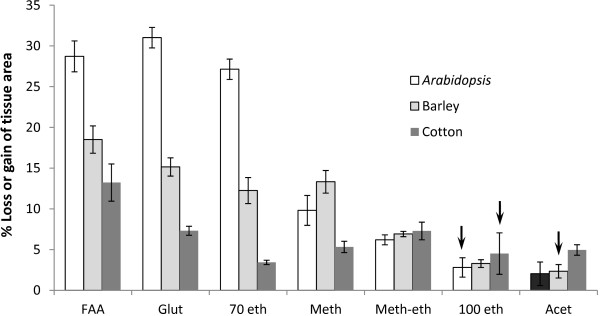
**Effect of SEM processing on dimensions of *A. thaliana*, cotton and barley leaf tissue.** Loss or gain in tissue area after processing through to critical point drying is expressed as percentage of original (fresh) tissue area. Treatments: FAA = FAA fixation; Glut = 3% glutaraldehyde; 70 eth = 70% ethanol fixation; Meth = Methanol fixation and dehydration followed by critical point drying in methanol; Meth-eth = methanol fixation followed by ethanol dehydration; 100 eth = absolute ethanol fixation; Acet = acetone fixation followed by ethanol dehydration (details in text). Bars are standard errors, 10 replicate tissue pieces were processed for glutaraldehyde, FAA, 70% ethanol and the 2 methanol treatments, and 16-20 replicates were processed for the absolute ethanol and acetone treatments. Only acetone treatment of *A. thaliana* leaf pieces resulted in overall tissue swelling (i.e., an average positive value), indicated by a dark grey-filled bar. Arrows indicate treatments in which some replicates swelled and others shrank, giving large standard errors.

Cotton leaves generally showed the least change in area after any fixation protocol, followed by barley and *A. thaliana* (Figure 1). For each species, a different protocol gave optimal results, for example, 70% ethanol fixation was best for cotton, and 100% ethanol best for barley. However, although 100% ethanol and acetone appeared preferable to the methanol-ethanol fixation for *A. thaliana*, the latter protocol was most reliable, indicated by the low standard errors in Figure 1. The raw data show that replicate samples fixed in 100% ethanol or acetone could either swell or shrink (Additional file 1 and Additional file 2).

**Figure 2:**
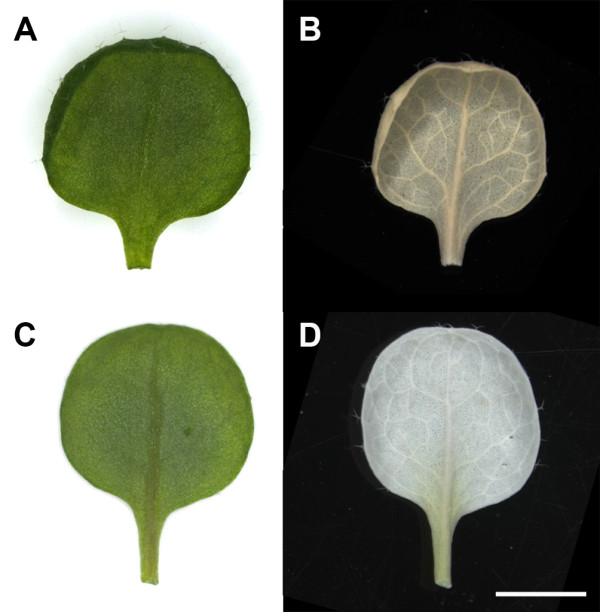
**Effect of SEM processing on *A. thaliana *leaf dimensions.** Visual comparison of glutaraldehyde and methanol-ethanol **(C,D)** fixation procedures, showing before **(A,C)** and after **(B,D)** critical point drying. Some leaves were slightly curled at the margins **(A)**; this curling did not seem to change after processing **(B)**, which was the same for curled leaves processed through all other fixative regimes. All images are at the same magnification. Scale bar = 2 mm, shown in **D**.

In all three species, glutaraldehyde or FAA fixation resulted in the most tissue shrinkage, and for *A. thaliana* and barley, 70% ethanol fixation gave similarly poor results. The procedure which consistently resulted in less than 8% shrinkage for all three species was fixation in methanol, followed by transfer to ethanol for 1 h then CPD (Figure 1). A visual comparison of *A. thaliana* leaves prepared by glutaraldehyde fixation and methanol fixation-ethanol dehydration shows the reduction in leaf area after glutaraldehyde fixation and CPD compared to fresh tissue (Figure 2).

Note that absolute ethanol, acetone and methanol rapidly penetrated tissues, causing both *A. thaliana* and barley leaf tissue to sink immediately without vacuum treatment. In comparison, tissues sank after light vacuum treatment in 70% ethanol, or only after extensive vacuum treatment in glutaraldehyde and FAA fixatives. An advantage of the methanol-ethanol treatment is that tissue can be left uncut since solvent penetration is very quick; for example, *A. thaliana* leaves were only cut at the petiole, while barley and cotton leaves were cut on four sides to fit the critical point drying basket.

### Effect of fixation methods on epidermal cell morphology

**Figure 3:**
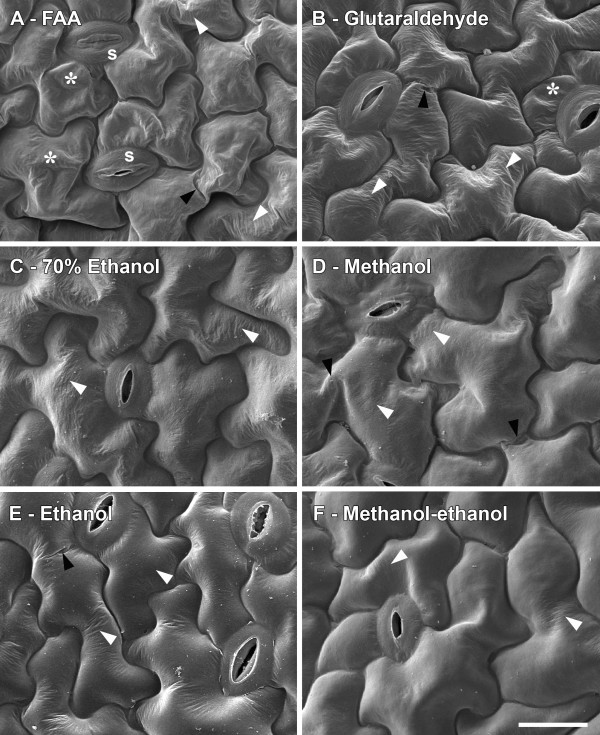
**Effect of SEM processing on morphology of *A. thaliana *leaf epidermal cells. A**, FAA fixation; **B**; Glutaraldehyde fixation; **C**, 70% ethanol fixation; **D**, methanol fixation followed by critical point drying in methanol; **E**, 100% ethanol fixation; and **F**, methanol fixation followed by ethanol dehydration and critical point drying in ethanol (details in text). Acetone fixation followed by ethanol dehydration showed similar preservation of morphology to 100% ethanol fixation. Stars indicate partial cell collapse, black arrowheads show cell wall folding and white arrowheads indicate cell wall wrinkles. s = stomata. All images are at the same magnification. Scale bar = 20 μm, shown in **F**.

Effects on tissue morphology generally reflected effects on tissue dimensions, as seen in Figure 3, in which methods causing the most to least changes to surface morphology are presented sequentially from Figure 3A-F. Using *A. thaliana* as an example since this tissue was more sensitive to the different fixation procedures than cotton or barley, we saw that the most damaging method was FAA fixation, which resulted in partial cell collapse, folding and wrinkling of walls (Figure 3A). Stomatal pores were also closed and wrinkled. There was a spectrum of similar artefacts following the other fixation procedures, however, solvent fixed-tissues (Figure 3C-F) appeared to fare better than those fixed in FAA (Figure 3A) or glutaraldehyde (Figure 3B). Of the solvent-based procedures, methanol fixation followed by ethanol dehydration then CPD in ethanol (Figure 3F), resulted in the least cell wall wrinkling, with negligible cell collapse or wall folding. The effects of the different treatments on morphology of barley and cotton leaves (Additional file 3), were similar to those observed in *A. thaliana* leaf surfaces, but were generally less marked.

**Figure 4:**
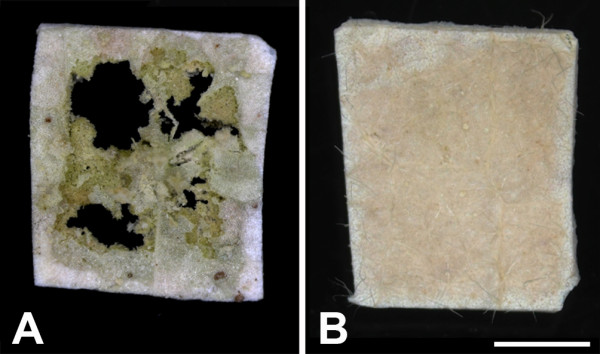
**Effect of dehydration time on cotton leaf pieces.** Pieces were fixed in methanol and dehydrated in ethanol for either 1 h **(A)** or overnight **(B)** before CPD. Images are at the same magnification. Scale bar = 2 mm, shown in **B**.

The tissues analysed here were all of similar area, although barley and cotton leaves were cut before processing, whereas *A. thaliana* leaves were processed whole. We examined the effect of tissue size by processing larger cotton leaf pieces using the methanol-ethanol fixation method outlined above, but they collapsed and were destroyed in the CPD vessel (Figure 4A). These larger leaf pieces remained intact if left to dehydrate in 100% dry ethanol overnight (Figure 4B), indicating that larger tissues can be processed using this protocol with some modifications to ensure more complete dehydration before CPD.

## Discussion

Our results reinforce previous observations that optimal SEM processing protocols differ for different tissues. Nevertheless, we found that of all the protocols tested, simultaneous fixation and dehydration in methanol followed by further ethanol dehydration (modified from [10]) gave the most consistent preservation of tissue dimensions and surface morphology of critical point dried plant tissues. Furthermore, the methanol-ethanol protocol is relatively non-toxic, quick and simple, and requires standard SEM preparation equipment.

We speculate that the differences in response of different tissues might arise from differences in leaf structure, epidermal surface coatings (wax and silica deposits) or wall composition between the three species. In addition, dimensional changes during specimen preparation are likely to be unequal in different parts of the tissue [7]. Since barley leaf epidermal cells are ordered in rows, this tissue might resist shrinkage in one direction, whereas there is no such directional organization of cells in cotton or *A. thaliana* leaves.

Methanol is a highly polar solvent similar in structure to water; it rapidly penetrates tissues and might simply replace water throughout the tissues. Even the walls of plant cells comprise up to 70% water by volume [17,18]. Hence, these properties of methanol make it an ideal fixative for SEM, since removal of water is critical for good preservation. Further dehydration with ethanol appears to be important, perhaps to completely remove water or to serve as a better intermediate solvent for CPD. The other solvents used as primary fixatives (ethanol and acetone) caused highly variable tissue shrinkage or swelling and their use is not recommended, unless thoroughly tested for a given tissue.

Glutaraldehyde has proven to be a good fixative for structural preservation for light microscopy and TEM, but as shown here and by others [10], is not necessarily a good fixative for SEM preparation. Overall, glutaraldehyde and FAA resulted in relatively poor preservation of tissue dimensions and morphology, which might reflect the different action of these fixatives and longer processing times required for these methods. Although these methods could be modified for different tissues to improve preservation, the methanol-ethanol protocol was more consistent and resulted in better morphological preservation.

A general protocol for methanol-ethanol fixation for SEM is outlined below:

1. Fix tissue in 100% methanol for 10 min or longer. Vacuum infiltrate if the tissue does not sink.

2. Transfer to 100% dry ethanol (30 min).

3. Dehydrate further in 100% dry ethanol for 30 min (small tissues) or overnight (large tissues), with further changes into fresh 100% ethanol if required.

4. Critical point dry following the manufacturer’s recommendations.

5. Mount tissue on SEM stub, coat with conducting metal if necessary (depending on type of imaging required e.g., no coating, carbon, or gold coating for Variable-Pressure SEM or gold for conventional high-vacuum SEM) and observe.

6. Before and after SEM observation, store in low-humidity environment, e.g. desiccator or controlled humidity cabinet.

## Conclusions

We found methanol to be a superior fixative and initial dehydrating agent for SEM processing of plant tissues. As noted earlier [10] and expanded on here, methanol fixation and dehydration followed by further ethanol dehydration resulted in better preservation of surface morphology and most consistent preservation of tissue dimensions compared to other solvent-based and aqueous fixation procedures. As with all procedures, optimal fixation and dehydration times should be empirically determined for a particular tissue type and size.

## Methods

### General methods

Relatively flat leaves from 2-3 week old agar-grown *Arabidopsis thaliana* (L.) Heynh (Columbia) seedlings were cut at the petiole. Leaves were harvested from young barley (*Hordeum vulgare* L., Golden promise variety) or cotton (*Gossypium hirsutum* L.; Coker variety) plants and 2-3 mm pieces were cut from the middle of the leaf with a sharp double-edged razor blade. Leaves or leaf pieces were imaged on a Leica MZFLIII dissecting microscope within 1 min, then transferred to fixative solution. Ten to 20 replicate samples were processed for each fixative regime. After fixation and dehydration steps (different methods outlined below), tissue was critical point dried in an Autosamdri-815 automatic critical point drier (Tousimis Research Corporation, Rockville USA) with a 20 min purge time. Tissue was imaged again under the dissecting microscope within an hour of completion of CPD. Images of fresh and post-critical point dried tissue were converted to greyscale and thresholded in Fiji (Image J version 1.47 h; [19]) for analysis of tissue area. To determine how the different preparation methods affected tissue morphology, 6 replicate critical point dried leaves or leaf pieces were mounted on aluminium stubs with double-sided sticky carbon tabs (ProSciTech, Qld, Australia), coated with gold (~20 nm) using an Emitech K500X sputter coater (Quorum Emitech, Kent, UK) and imaged in a Zeiss EVO LS 15 Scanning Electron Microscope at 15 kV accelerating voltage. Two images were taken from each replicate.

### Fixation and dehydration protocols

#### FAA

Leaf tissue was fixed according to Bomblies et al. (2008) [20]. Tissue pieces were immersed in FAA fixative (3.7% v/v formaldehyde, 50% ethanol, 5% acetic acid) and subjected to a light vacuum until the tissue pieces sank. Tissue was then fixed overnight (approximately 18 h) at 4°C. Tissue was rinsed 3 times in 25 mM sodium phosphate buffer (pH 7) before dehydrating in an ethanol series (30%, 50%, 70%, 95% and 100% dry, 30 min each step). 100% dry ethanol was changed twice and the tissue was stored overnight at 4°C before CPD the next day. Note that this protocol calls for an osmium tetroxide post-fixation step before dehydration, which reduces tissue charging when observed in the SEM. However, we found that this step was generally unnecessary if the tissue was gold-coated before SEM imaging, so the osmium post-fixation step was omitted.

#### Glutaraldehyde

Leaf tissue was immersed in standard fixative for SEM (e.g., [3,8]) consisting of 3% glutaraldehyde in 25 mM sodium phosphate buffer (pH 7) and with 0.01% Triton X-100 to improve penetration of fixative. For the first 10-20 min of fixation, tissue was vacuum infiltrated with fixative plus detergent until the tissue sank. After the tissue had sunk this was replaced with fixative minus detergent and left overnight at 4°C. The tissue was then washed 3 × 10 min in 25 mM sodium phosphate buffer, rinsed in distilled water and dehydrated through an ethanol series in 10% increments, starting at 10%, 30 min each step. Once in 100% dry ethanol, this was changed twice, 30 min each, then the tissue was left overnight in 100% dry ethanol at 4°C before CPD the following day.

#### 70% ethanol

Leaf tissue was immersed in 70% ethanol for 1 h, with vacuum infiltration within the first 5 min or until the tissue sank, if necessary, then dehydrated to 100% dry ethanol in 10% steps, 30 min each step. The 100% dry ethanol was changed twice, tissue was left overnight in 100% dry ethanol at 4°C then critical point dried the next day.

#### Methanol

This method is based on Neinhuis and Edelmann (1996) [10]. Leaf tissue was immersed in 100% dry methanol for 10 min, followed by 2 × 30 min changes in 100% dry methanol; vacuum infiltration was not necessary as tissue sank immediately. Tissue was then critical point dried immediately with methanol as the transitional fluid.

#### Methanol-ethanol

This method is based on Neinhuis and Edelmann (1996) [10]. Leaf tissue was immersed in 100% dry methanol for 10 min, followed by 2 × 30 min changes in 100% dry ethanol (vacuum infiltration was not needed). Tissue was then critical point dried immediately with ethanol as the transitional fluid. To explore this method further we fixed larger cotton leaf pieces as described above. One lot of tissue was then critical point dried immediately as above, another lot was transferred into fresh dry ethanol and left overnight at 4°C before critical point drying the next day.

#### 100% ethanol

Leaf tissue was immersed in dry 100% ethanol for 10 min, followed by 2 × 30 min changes in 100% dry ethanol (vacuum infiltration was not needed). The tissue was then critical point dried in 100% dry ethanol.

#### Acetone

Leaf tissue was immersed in dry acetone for 10 min, followed by 2 × 30 min changes in 100% dry ethanol (vacuum infiltration was not needed). The tissue was then critical point dried in 100% dry ethanol. The tissue was transferred to ethanol since acetone is not recommended for use in the Autosamdri-815 critical point dryer.

## Competing interests

The authors declared that they have no competing interests.

## Authors’ contributions

MT contributed to conception and design of the study, carried out all experiments and analysis and drafted the manuscript. RW contributed to conception and design of the study and drafted the manuscript. Both authors read and approved the final manuscript.

## Supplementary Material

Additional file 1 **Effect of absolute ethanol fixation and dehydration and critical point drying on *A. thaliana* leaf area; raw data used for graph in Figure **1**.** ‘Fresh’ refers to area (mm^2^) of fresh tissue, while ‘CPD’ refers to area of tissue after critical point drying. Also shown is the % loss (indicated by negative values) or gain in area after processing. Click here for file

Additional file 2 **Effect of 100% acetone fixation, ethanol dehydration, and critical point drying in ethanol, on *A. thaliana* leaf area; raw data used for graph in Figure **1**.** ‘Fresh’ refers to area (mm^2^) of fresh tissue, while ‘CPD’ refers to area of tissue after critical point drying. Also shown is the % loss (indicated by negative values) or gain in area after processing. Click here for file

Additional file 3 **Effect of SEM processing on morphology of barley (A, B) and cotton (C, D) leaf epidermal cells, processed by FAA (A, C) or methanol-ethanol (B, D) fixation.** Stars indicate partial cell collapse, white arrowheads indicate cell wall wrinkles. s = stomata. All images are at the same magnification. Scale bar = 30 μm, shown in D. Click here for file

## References

[B1] Beckett A Read ND Porter R Variations in fungal spore dimensions in relation to preparatory techniques for light microscopy and scanning electron microscopy J Microsc 1984 136 1 87 95 10.1111/j.1365-2818.1984.tb02548.x

[B2] Boyde A Boyde S Further studies of specimen volume changes during processing for SEM: including some plant tissue Scan Electron Microsc 1980 II 117 124 6999595

[B3] Moncur MW Shrinkage of plant material during critical point drying Scanning 1979 2 175 177 10.1002/sca.4950020309

[B4] Parsons E Bole B Hall DJ Thomas DE A comparative survey of techniques for preparing plant surfaces for the scanning electron microscope J Microsc 1974 101 1 59 75 10.1111/j.1365-2818.1974.tb03867.x

[B5] Boyde A Maconnachie E Volume changes during preparation of mouse embryonic tissue for scanning electron microscopy Scanning 1979 2 149 163 10.1002/sca.4950020305

[B6] McCully ME Canny MJ Huang CX Cryo-scanning electron microscopy (CSEM) in the advancement of functional plant biology. Morphological and anatomical applications Funct Plant Biol 2009 36 97 124 10.1071/FP08304 32688631

[B7] Read ND Porter R Beckett A A comparison of preparative techniques for the examination of the external morphology of fungal material with the scanning electron microscope Can J Bot 1983 61 2059 2078 10.1139/b83-223

[B8] Pathan AK Bond J Gaskin RE Sample preparation for scanning electron microscopy of plant surfaces–horses for courses Micron 2008 39 1049 1061 10.1016/j.micron.2008.05.006 18586502

[B9] Sargent JA The preparation of leaf surfaces for scanning electron microscopy: a comparative study J Microsc 1983 129 1 103 110 10.1111/j.1365-2818.1983.tb04164.x

[B10] Neinhuis C Edelmann HG Methanol as a rapid fixative for the investigation of plant surfaces by SEM J Microsc 1996 184 1 14 16 10.1046/j.1365-2818.1996.d01-110.x

[B11] Schwab B Hulskamp M Quick and easy fixation of plant tissues for scanning electron microscopy Cold Spring Harb Protoc 2010 8 1 doi:10.1101/pdb.prot4934 10.1101/pdb.prot4934 20679373

[B12] Das K Baruah KK Methane emission associated with anatomical and morphophysiological characteristics of rice (*Oryza sativa*) plant Physiol Plant 2008 134 303 312 10.1111/j.1399-3054.2008.01137.x 18507814

[B13] Hiscock SJ Hoedemaekers K Friedman WE Dickinson HG The stigma surface and pollen-stigma interactions in *Senecio squalidus* L. (Asteraceae) following cross (compatible) and self (incompatible) pollinations Int J Plant Sci 2002 163 1 1 16

[B14] Melzer B Steinbrecher T Seidel R Kraft O Schwaiger R Speck T The attachment strategy of English ivy: a complex mechanism acting on several hierarchical levels J R Soc Interface 2010 7 1383 1389 10.1098/rsif.2010.0140 20462880 PMC2894893

[B15] Prum B Seidel R Florian Bohn H Speck T Impact of cell shape in hierarchically structured plant surfaces on the attachment of male Colorado potato beetles (*Leptinotarsa decemlineata*) Beilstein J Nanotechnol 2012 3 57 64 22428097 10.3762/bjnano.3.7 PMC3304315

[B16] Zelko I Luz A Sterckeman T Martinka M Kollarova K Liskova D An easy method for cutting and fluorescent staining of thin roots Ann Bot 2012 110 475 478 10.1093/aob/mcs046 22419758 PMC3394640

[B17] O’Neill MA York WS Rose JKC The composition and structure of plant primary cell walls The Plant Cell Wall. Annual Plant Reviews, volume 8 2003 UK: Blackwell Publishing Ltd 3

[B18] Edelmann HG Characterization of hydration-dependent wall-extensible properties of rye coleoptiles: evidence for auxin-induced changes of hydrogen bonding J Plant Physiol 1995 145 491 497 10.1016/S0176-1617(11)81776-1

[B19] Fiji is Just ImageJ http://fiji.sc/Fiji

[B20] Bomblies K Shukla V Grajam C Scanning Electron Microscopy (SEM) of plant tissues Cold Spring Harb Protoc 2008 3 4 1 3

