# Supplementary material for: Methanol fixation of plant tissue for Scanning Electron Microscopy improves preservation of tissue morphology and dimensions
Source: Plant Methods. 2013 Oct 2;9:36. doi: 10.1186/1746-4811-9-36 (PMC3853006; doi:10.1186/1746-4811-9-36)
Supplement: Additional file 1 — Effect of absolute ethanol fixation and dehydration and critical point drying on A. thaliana leaf area; raw data used for graph in Figure 1. ‘Fresh’ refers to area (mm2) of fresh tissue, while ‘CPD’ refers to area of tissue after critical point drying. Also shown is the % loss (indicated by negative values) or gain in area after processing. [file 1746-4811-9-36-S1.pdf]

| Replicate | Fresh | CPD | % loss or gain |
|---|---|---|---|
| 1 | 9.57 | 9.72 | 1.58 |
| 2 | 8.72 | 8.61 | -1.23 |
| 3 | 10.87 | 10.71 | -1.53 |
| 4 | 9.06 | 9.10 | 0.45 |
| 5 | 11.70 | 11.87 | 1.50 |
| 6 | 8.76 | 9.07 | 3.50 |
| 7 | 9.13 | 9.21 | 0.85 |
| 8 | 10.25 | 10.63 | 3.76 |
| 9 | 9.58 | 10.04 | 4.78 |
| 10 | 10.43 | 10.40 | -0.29 |
| 11 | 6.92 | 6.34 | -8.45 |
| 12 | 7.66 | 7.24 | -5.55 |
| 13 | 7.02 | 6.23 | -11.27 |
| 14 | 5.21 | 5.04 | -3.43 |
| 15 | 7.92 | 7.05 | -11.01 |
| 16 | 7.22 | 6.33 | -12.33 |
| 17 | 7.34 | 6.92 | -5.72 |
| 18 | 7.65 | 7.10 | -7.21 |
| 19 | 7.73 | 7.53 | -2.60 |
| 20 | 8.38 | 8.19 | -2.20 |
| **mean** | 8.56 | 8.37 | 2.82 |
| **SE** | 0.36 | 0.42 | 1.19 |
