# Supplementary figures and images for: Methanol fixation of plant tissue for Scanning Electron Microscopy improves preservation of tissue morphology and dimensions
Source: Plant Methods. 2013 Oct 2;9:36. doi: 10.1186/1746-4811-9-36 (PMC3853006; doi:10.1186/1746-4811-9-36)

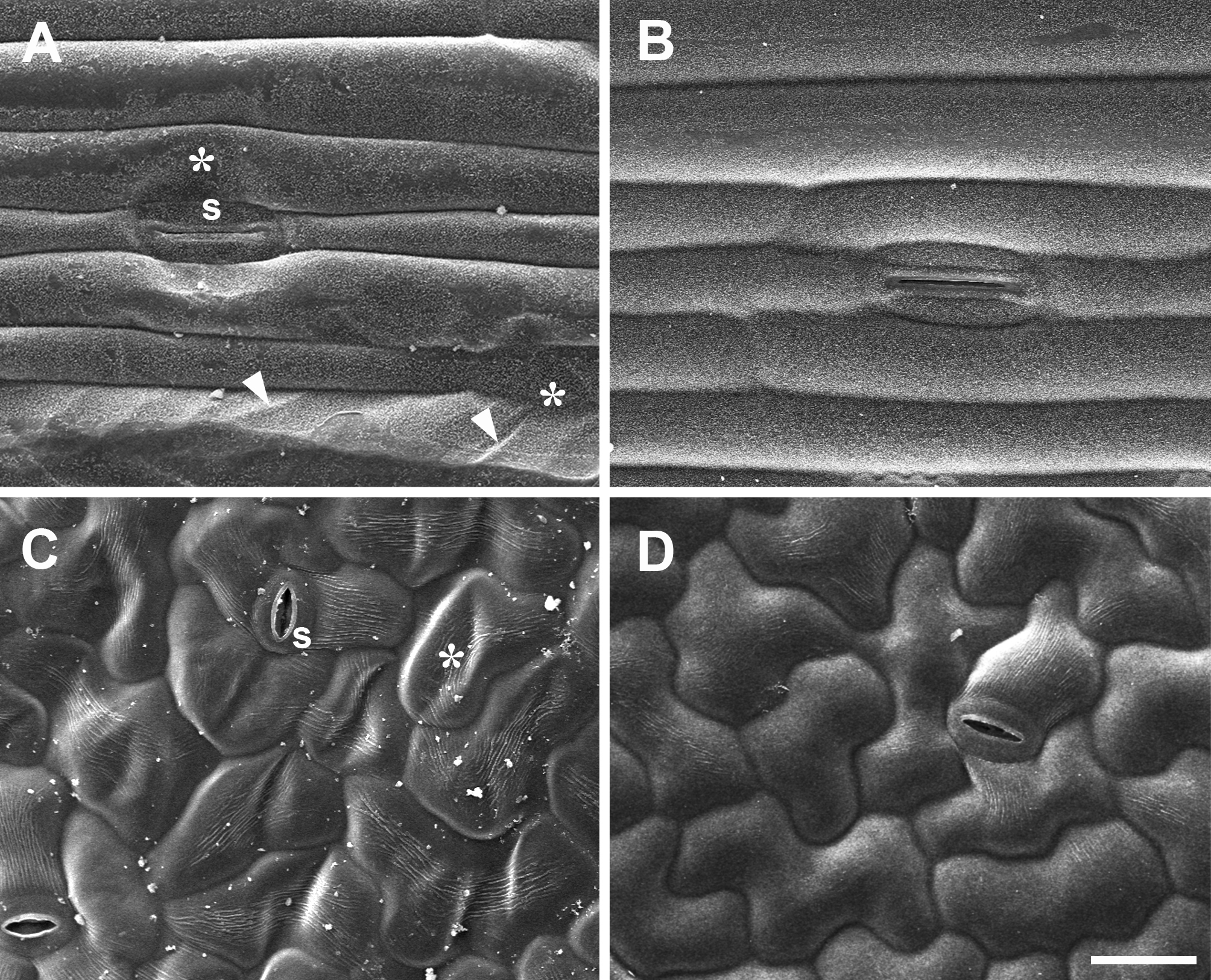

Supplement: Additional file 3 — Effect of SEM processing on morphology of barley (A, B) and cotton (C, D) leaf epidermal cells, processed by FAA (A, C) or methanol-ethanol (B, D) fixation. Stars indicate partial cell collapse, white arrowheads indicate cell wall wrinkles. s = stomata. All images are at the same magnification. Scale bar = 30 μm, shown in D. [file 1746-4811-9-36-S3.jpeg]
